# Door of Perception: NIEHS Portal Shows Way to Better Disaster Response

**Published:** 2007-04

**Authors:** Charles W. Schmidt

Hurricane Katrina—which killed 1,300 people, disrupted the lives of 650,000, and produced an estimated $125 billion in recovery and reconstruction costs—brought the need for better disaster response into sharp focus. In this issue, researchers introduce the NIEHS Portal, a state-of-the-art web-based system to improve decision making during disaster response **[*EHP* 115:564–571; Pezzoli et al.]**.

The portal was designed to fulfill three objectives: to monitor disaster-related human and environmental health impacts; to assess and reduce pollutant exposures caused by disasters; and to develop science-based recovery strategies. The portal does this by combining geographically referenced data on roads, power plants, contaminant release sources, flood measures, and local demography in a cyber-infrastructure called “Telescience,” which was developed at the University of California, San Diego. This cyberinfrastructure lets users share computer power and storage over the Internet.

A user-friendly interface provides access to project-relevant databases and data integration tools. High-speed network connections allow researchers to use supercomputing facilities and massive data storage sites as if they were on their desktop. A geographic information system (GIS) manages the data, and an accessible interface allows users to contribute new information and participate in online discussions and collaborative workspaces. Contributors are responsible for the accuracy of the data they provide. Access to data associated with any given research project is governed by the members of that research group.

In its current deployment, the portal assembles GIS data for Texas, Louisiana, and Mississippi, and includes high-resolution data layers for the regions that were affected by Hurricanes Katrina and Rita in 2005. Scientists have begun exploring ways to use the system. One project supports ongoing efforts to study and mitigate the health effects of flood-induced indoor mold, particularly asthma among exposed children. Scientists link high mold concentrations with population information to identify locations of potential high exposures. The portal is also supporting studies of toxic sediments, particularly hot spots generated by the release of contaminants such as sewage and industrial chemicals during the storms.

The authors stress how the portal can also address the research needs of exposure biology and gene–environment interactions. The system’s massive computing power can integrate population-level studies of genetics with real-time exposure monitoring and environmental sampling, advancing the NIEHS’s goal of studying molecular processes in environmental health.

## Figures and Tables

**Figure:**
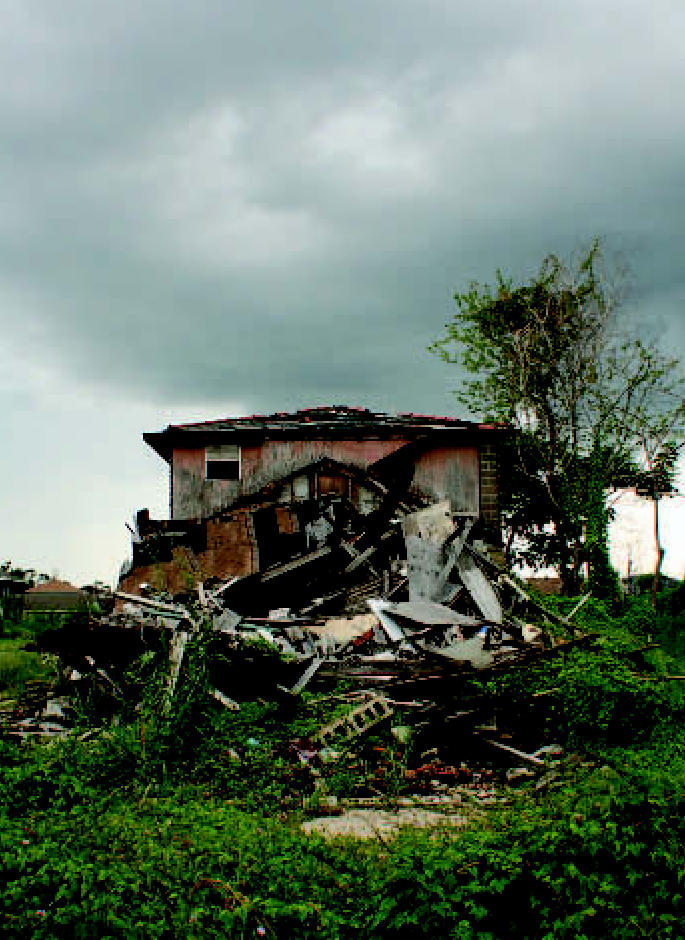
SOS Nineteen months after Katrina, Gulf Coast residents are still in the storm’s shadow.

